# The Combined Risk of Marfan Syndrome and Bicuspid Aortic Valve in the Elderly

**DOI:** 10.7759/cureus.81314

**Published:** 2025-03-27

**Authors:** Regina McPherson, Marina Shehata, Guillermo Izquierdo-Pretel

**Affiliations:** 1 Anatomical Sciences, American University of Antigua, St. John's, ATG; 2 Internal Medicine, Florida International University, Herbert Wertheim College of Medicine, Miami, USA

**Keywords:** aortic stenosis, bicuspid aortic valve, marfan syndrome, thoracic aortic aneurysm, transcatheter aortic valve replacement

## Abstract

Marfan syndrome is a genetic condition that causes connective tissue abnormalities that can ultimately lead to cardiovascular complications such as aneurysms. When a bicuspid aortic valve is also present, which is a congenital heart defect characterized by the aortic valve having two cusps instead of three, a combined effect of serious cardiovascular complications can result. There is an additive and increased risk of aortic complications due to the combined impact on aortic integrity that can lead to aneurysm formation and/or dissections. A severe case of aortic stenosis with a thoracic aortic aneurysm (TAA) was discovered in a patient found to have Marfan syndrome. An elderly female patient, without any relevant medical history, was evaluated for progressive dyspnea following the death of her spouse. Upon examination, the patient exhibited characteristics of Marfan syndrome. Subsequent imaging revealed a bicuspid aortic valve and TAA. A multidisciplinary approach was necessary, involving various specialties to guide treatment. Given the patient’s age and high-risk profile, she was not a candidate for TAA repair. However, the patient underwent a transcatheter aortic valve replacement (TAVR) with medical management and routine follow-ups. Awareness of the heightened risks associated with a bicuspid aortic valve and Marfan syndrome is crucial, as early intervention can mitigate complications and improve the quality of life.

## Introduction

Marfan syndrome and bicuspid aortic valve are significant medical conditions that affect the cardiovascular system. The presence of both conditions, especially in the elderly, can lead to serious aortic complications, including aneurysms, dissections, and aortic regurgitation. Marfan syndrome is a genetic disorder caused by a mutation in the FBN1 gene, which affects connective tissue throughout the body [1]. It is one of the most common genetic abnormalities of connective tissues, typically presenting with cardiovascular, ocular, and musculoskeletal abnormalities. The weakened connective tissue makes the aorta more susceptible to dilatation and dissection. Patients with Marfan syndrome often exhibit characteristic skeletal abnormalities, including dolichostenomelia, arachnodactyly, thoracolumbar scoliosis, and pectus deformities [2]. In addition, other conditions associated with Marfan syndrome include pneumothorax and abnormally indented hip sockets [3]. Early diagnosis is typically based on skeletal abnormalities or ocular findings. However, cardiovascular complications are the major cause of morbidity and mortality if undiagnosed and untreated. Aortic root dilations can progress to dissections with an acute presentation.

A bicuspid aortic valve is a congenital heart defect characterized by the presence of two cusps instead of the normal three. Clinicians use the Sievers classification system to describe the three different types of bicuspid aortic valve and to help assess the level of surgical risk during treatment. Type 0 valves describe two symmetrical leaflets with no raphe or ridges; type 1 valves describe one raphe where two leaflets have fused; and type 3 valves describe the presence of two raphes. The exact mechanism remains unclear, with some theories suggesting abnormal blood flow across the valve during the developmental phase, resulting in incomplete cusp separation [4]. The abnormal valve structure leads to turbulent blood flow and increased stress on the aortic wall, and the most frequently associated abnormality is a dilated thoracic aorta. BAV is the most common congenital cardiac abnormality, with a prevalence of 1-2%. The prevalence increases to greater than 20% when in conjunction with coarctation of the aorta. Presentation can vary, ranging from severe aortic stenosis in childhood to an asymptomatic state until adulthood. Middle-aged patients often present with fibrocalcific stenosis requiring surgical intervention [5].

Despite their distinct etiologies, both conditions share a commonality in their potential to cause serious cardiovascular complications if left untreated. Patients with both Marfan syndrome and a bicuspid aortic valve have an increased risk of aortic complications up to 5% due to the combined effect on aortic integrity [5]. These patients are predisposed to developing larger aortic aneurysms at a younger age. Given the heightened risk of severe complications, a multidisciplinary approach and close monitoring are crucial to improving outcomes and reducing the risk of serious aortic complications. A comprehensive, interdisciplinary approach tailored to the patient’s needs can significantly enhance outcomes and quality of life.

This study illustrates the challenges in managing an elderly patient with Marfan syndrome and BAV complicated by severe aortic stenosis as well as a thoracic aortic aneurysm and highlights the importance of a multidisciplinary approach.

## Case presentation

A 74-year-old female with no significant past medical history presented to the emergency room with complaints of intermittent sharp chest pain radiating to the right chest, accompanied by shortness of breath, cough, palpitations, and orthopnea that began three weeks prior. She reported progressively worsening symptoms over the past three days, with her chest pain intensifying from 2/10 to 4/10 with a Canadian Cardiovascular Society (CCS) grading between II and III. The patient noted the onset of these symptoms following the passing of her husband. She denied any history of thyroid abnormalities, asthma, seizures, cerebrovascular accidents (CVA), chronic obstructive pulmonary disease (COPD), smoking, coronary artery disease (CAD), myocardial infarction (MI), or cardiac arrhythmias. Additionally, she reported no lower extremity edema, weight gain, or near-syncope. The patient was unaware of any health conditions in her family, including cardiovascular disease or genetic conditions. She was a lifetime non-smoker and did not do illicit drugs. The patient has a history of working at a garbage bag manufacturing factory for 20 years but wore a mask. She is now retired.

**Figure 1:**
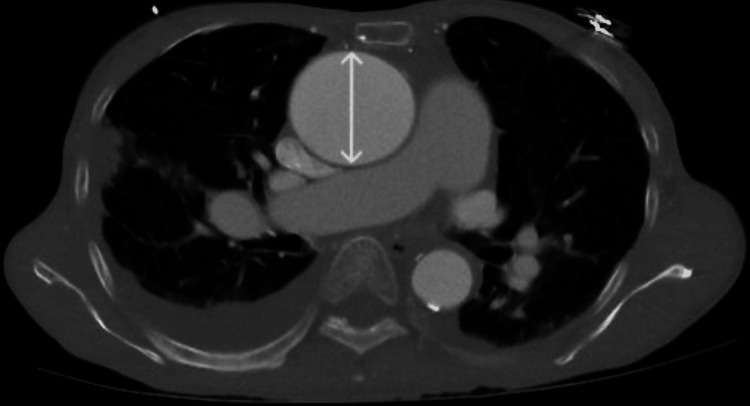
An ascending thoracic aortic aneurysm measuring 5.4 x 4.5 cm (arrow).

**Figure 2:**
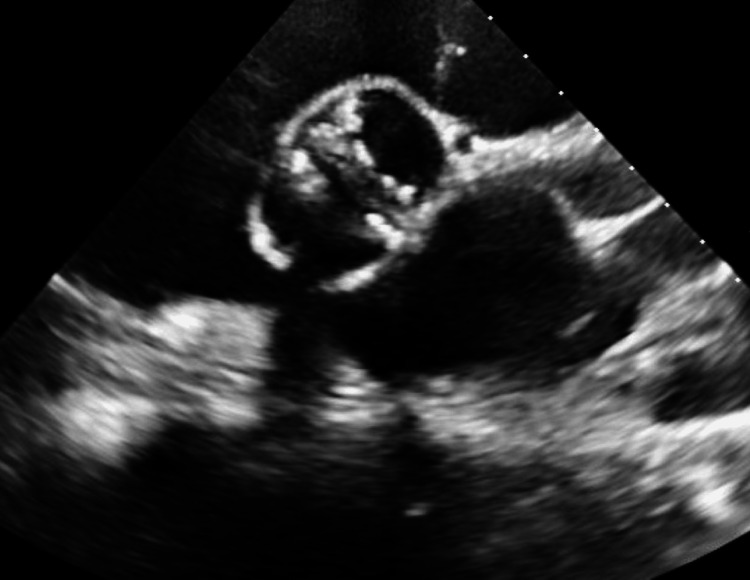
Bicuspid aortic valve with severe valvular aortic stenosis.

In the emergency room, the patient was found to be in atrial fibrillation with a rapid ventricular rate (RVR), a heart rate of 101 beats per minute on telemetry, and a blood pressure of 84/68 mmHg, with oxygen saturation of 99% on a 2 L nasal cannula. Laboratory tests revealed a slightly elevated troponin level of 0.049 ng/mL, elevated liver function tests (LFTs), and a N-terminal pro-brain natriuretic peptide (proBNP) level of 14,600 pg/mL as seen in Table 1. A computed tomography angiography (CTA) showed bilateral pleural effusions, bronchiectasis in the upper lobes, and an ascending thoracic aortic aneurysm measuring 5.4 x 4.5 cm (Figure 1). There was also evidence of coronary calcifications in the left anterior descending (LAD) and left circumflex arteries. An electrocardiogram (EKG) showed sinus rhythm with occasional premature atrial contractions (PACs) and non-specific T-wave abnormalities. An echocardiogram (ECHO) revealed an ejection fraction (EF) of 25-30%, severely reduced left ventricular systolic function, borderline reduced right ventricular systolic function, a bicuspid aortic valve with low flow, low gradient severe valvular aortic stenosis, and moderate aortic root dilatation (Figure 2). The aortic mean gradient was measured at 41.0 mmHg, with an aortic valve area of 0.41 cm^2^. The patient was admitted for stabilization of heart failure with subsequent evaluation for aortic valve replacement and repair of the thoracic aortic aneurysm.

**Table 1:** Laboratory values on admission. BUN: blood urea nitrogen; AST: aspartate aminotransferase; ALT: alanine aminotransferase; Pro-BNP: N-terminal pro-brain natriuretic peptide

Lab value	Results	Reference ranges
BUN	23 mg/dL	7-17 mg/dL
Creatinine	0.90 mg/dL	0.52-1.04 mg/dL
AST	112 units/L	15-46 units/L
ALT	143 units/L	9-30 units/L
Pro-BNP	14,600 pg/mL	0-125.0 pg/mL
Troponin	0.049 ng/mL	0.0-0.034 ng/mL

**Figure 3:**
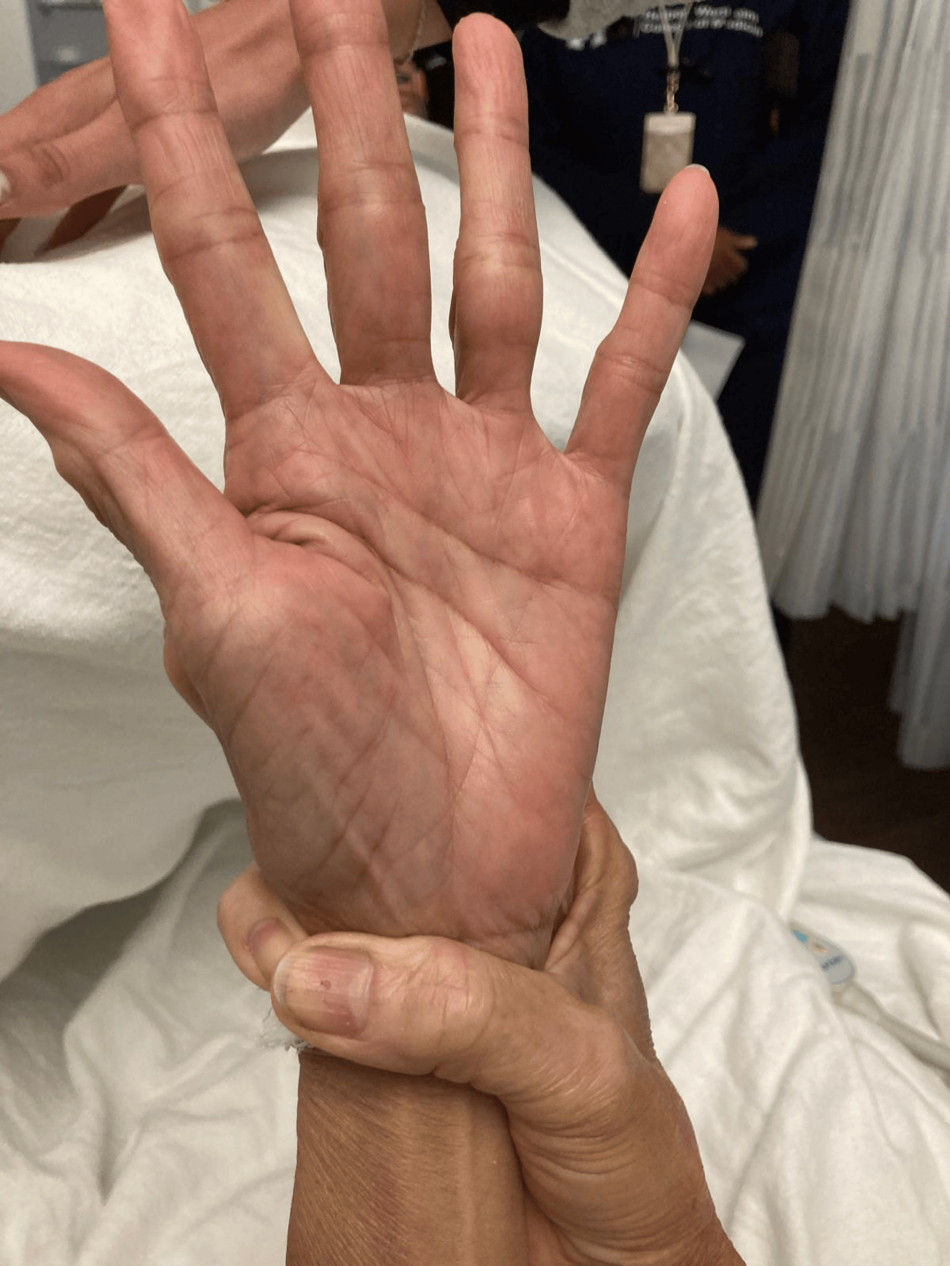
Positive Walker-Murdoch sign. The patient’s thumb easily connects with the fifth finger when encircling the wrist, suggesting joint hypermobility seen in Marfan syndrome.

Upon examination, the patient was noted to have multiple components of Marfan's syndrome with the following measurements: height 149 cm, weight 32.6 kg, arm span 158 cm. The arm span to height ratio was >1.05. The Walker-Murdoch wrist sign, where the thumb and fifth digit overlap when encircling the opposite wrist, was present as shown in Figure 3. It signifies arachnodactyly, which is a common skeletal manifestation of Marfan syndrome. The patient was also noted to have ectopia lentis. Per Ghent criteria, the patient meets the diagnosis of Marfan syndrome. Genetic testing was ordered; however, it was not completed. During the evaluation, the patient denied chest pain but reported orthopnea and dyspnea with exertion. Auscultation revealed a soft S2 crescendo-decrescendo systolic murmur heard best in the second right intercostal space. There was no evidence of jugular venous distention (JVD). A bedside point-of-care ultrasound (POCUS) demonstrated multiple B-lines throughout the bilateral lung fields. There was evidence of mild-to-moderate venous excess with inferior vena cava (IVC) noted to be <2, but abnormal pulse Doppler in the hepatic and portal veins.

An ischemic work-up was initiated and the patient underwent left and right cardiac catheterization, which revealed no evidence of obstructive coronary artery disease, normal right ventricular filling pressures, and no evidence of pulmonary hypertension. After a multidisciplinary meeting and discussion of the case with the cardiology team and cardiothoracic surgery team, the patient was scheduled for transcatheter aortic valve replacement (TAVR). The patient was not a candidate for thoracic aortic aneurysm (TAA) repair given her high-risk index. The patient underwent successful transfemoral transcatheter aortic valve replacement with a 23 mm (+2cc) SAPIEN S3 (Santa Ana, CA: Edwards Lifesciences) valve and required a temporary pacemaker implantation. There was concern for mild/moderate aortic insufficiency following valve replacement. The patient was noted to have a pericardial effusion that was increased from prior; however, it was stable on follow-up imaging. A plan was made to follow the TAA as an outpatient and to consider options once the patient’s left ventricular function improves. The patient was discharged with a cardiology follow-up appointment. 

## Discussion

When considering the additive effects of Marfan syndrome and bicuspid aortic valve in the elderly, we must aim to understand the individual implications of each condition and how they can synergistically impact cardiovascular health. The cumulative effects of these conditions over time can contribute to more severe aortopathy. Although both conditions are typically diagnosed earlier in life, their complications can become more pronounced with age. Bicuspid aortic valve is associated with aortic complications such as critical aortic dilation with an increased risk of dissection and rupture. Marfan syndrome is characterized by mutations in the fibrillin-1 gene, which causes abnormal elastic properties in the aorta which leads to decreased compliance and progressively increased dilation [6].

Patients with both Marfan syndrome and a bicuspid aortic valve present with unique challenges and risks; the presence of aortic root dilation in Marfan syndrome when combined with the hemodynamic changes associated with a bicuspid aortic valve exacerbates the risk of aortic dissection, aortic valve dysfunction, and TAA [7]. In fact, as individuals with both Marfan and bicuspid aortic valve age, there is increasing compromise of the structural integrity of the aorta and aortic valve. Marfan syndrome is characterized by progressive aortic root dilation with aging, and when this is added to the altered flow dynamics seen in patients with a bicuspid aortic valve, there is a resultant accelerated degeneration of the aortic valve and worsening aortic regurgitation and stenosis [6].

Aortic aneurysm formation due to cystic medial degeneration is another severe complication associated with both conditions. Marfan syndrome and bicuspid aortic valve both independently predispose patients to aortic aneurysms, and the associated combined effects in the elderly include increased risk of rupture and rapid expansion due to long-term stress on the aorta. These aneurysms can lead to catastrophic complications, including aortic dissection or rupture, and are hence termed “silent killers” as 95% of patients with TAA are asymptomatic [8]. Other risks include arrhythmias, such as an increased risk of atrial fibrillation. An aortic aneurysm repair is typically necessary. However, given this patient's age and her high-risk index, she was not a great candidate for a TAA repair. Studies show that an aneurysmal repair has a 30-day survival rate of 94% and 5- and 10-year survival rates after surgery of 75% and 56%, respectively [9].

Due to the presence of larger aortic dimensions in patients with Marfan syndrome and bicuspid aortic valve, there is often a trend towards surgical intervention at a younger age. Surgical intervention is recommended when the aortic diameter reaches 5 cm or exceeds 1 cm per year [10]. Surgical thresholds for aortic root replacement in Marfan syndrome are influenced by diameter, family history of aortic dissection, ascending aortic dilation, rapid growth, which are risk markers for aortic complications. Earlier interventions may be warranted given the combined risks with decisions based on aortic size, growth rate, and symptoms. With or without surgery, these patients are at high risk of decompensation and require continued surveillance [11]. Considering the adverse effects associated with valvulopathies, valve replacement is necessary for severe aortic stenosis or regurgitation [12]. Transcatheter aortic valve replacement (TAVR) may be considered in high-risk surgical candidates.

## Conclusions

In conclusion, the combination of Marfan syndrome and a bicuspid aortic valve significantly elevates cardiovascular risks, particularly in elderly patients. When these conditions are present together, their synergistic effect can lead to severe, life-threatening complications. Given this heightened risk profile, a multidisciplinary approach involving primary care physicians, cardiologists, and geneticists is essential for effective care. Regular follow-up, monitoring, and adherence to medical management are crucial. With comprehensive and proactive management strategies, it is possible to mitigate the risks associated with Marfan syndrome and a bicuspid aortic valve and improve the quality of life for elderly patients facing these cardiovascular challenges.
